# Peroxiredoxin 1 induces inflammatory cytokine response and predicts outcome of cardiogenic shock patients necessitating extracorporeal membrane oxygenation: an observational cohort study and translational approach

**DOI:** 10.1186/s12967-016-0869-x

**Published:** 2016-05-04

**Authors:** Chia-Hsiung Liu, Shuenn-Wen Kuo, Li-Ming Hsu, Shu-Chien Huang, Chih-Hsien Wang, Pi-Ru Tsai, Yih-Sharng Chen, Tzuu-Shuh Jou, Wen-Je Ko

**Affiliations:** Graduate Institute of Clinical Medicine, College of Medicine, National Taiwan University, Taipei, Taiwan, ROC; Department of Surgery, National Taiwan University Hospital, Taipei, Taiwan, ROC; Department of Traumatology, National Taiwan University Hospital, Taipei, Taiwan, ROC; College of Medicine, National Taiwan University, Taipei, Taiwan, ROC

**Keywords:** Extracorporeal membrane oxygenation, Cardiogenic shock, Peroxiredoxin 1, Danger-associated molecular patterns, Ischemia-reperfusion, Systemic inflammatory response syndrome

## Abstract

**Background:**

Extracellular peroxiredoxin 1 (Prdx1) has been implicated to play a pivotal role in regulating inflammation; however, its function in tissue hypoxia-induced inflammation, such as severe cardiogenic shock patients, has not yet been defined. Thus, the objective of this study was to test the hypothesis that Prdx1 possesses prognostic value and instigates systemic inflammatory response syndrome in cardiogenic shock patients undergoing extracorporeal membrane oxygenation (ECMO) support.

**Methods:**

We documented the early time course evolution of circulatory Prdx1, hypoxic marker carbonic anhydrase IX, inflammatory cytokines including IL-6, IL-8, IL-10, MCP-1, TNF-α, IL-1β, and danger signaling receptors (TLR4 and CD14) in a cohort of cardiogenic shock patients within 1 day after ECMO support. In vitro investigations employing cultured murine macrophage cell lines and human monocytes were applied to clarify the relationship between Prdx1 and inflammatory response.

**Results:**

Prdx1 not only peaked earlier than all the other cytokines we studied during the initial course, but also predicted a worse outcome in patients who had higher initial Prdx1 plasma levels. The Prdx1 levels in patients positively correlated with hypoxic markers carbonic anhydrase IX and lactate, and inflammatory cytokines. In vitro study demonstrated that hypoxia/reoxygenation induced Prdx1 release from human monocytes and enhanced the responsiveness of the monocytes in Prdx1-induced cytokine secretions. Furthermore, functional inhibition by Prdx1 antibody implicated a crucial role of Prdx1 in hypoxia/reoxygenation-induced IL-6 secretion.

**Conclusions:**

Prdx1 release during the early phase of ECMO support in cardiogenic shock patients is associated with the development of systemic inflammatory response syndrome and poor clinical outcomes. Thus, circulating Prdx1 provides not only prognostic information but may be a promising target against ischemia/reperfusion injury.

**Electronic supplementary material:**

The online version of this article (doi:10.1186/s12967-016-0869-x) contains supplementary material, which is available to authorized users.

## Background

Extracorporeal membrane oxygenation (ECMO) is an option for patients with refractory cardiogenic shock who would otherwise face fatal outcome. Although the use of ECMO is trending high worldwide, the associated mortality rate remains grave [1, 2]. Therefore, understanding the underlying mechanisms which contribute to poor clinical outcomes is a pivotal issue to improve patient selection as well as further refine this therapeutic intervention.

Cardiogenic shock, irrespective of the etiology, is a state of systemic hypoperfusion and global tissue ischemia [3]. Although ECMO restores oxygenated blood to the ischemic tissue, reperfusion paradoxically worsens the damages caused by ischemia alone in certain viable cells. This ischemia/reperfusion (I/R) injury can be imputed to a burst of oxidative stress and development of a systemic inflammatory response syndrome (SIRS), which is associated with multisystem organ failure (MOF) and considerable mortality [4]. Activation of innate immunity is intimately involved in the deleterious SIRS following I/R [5–8]. Toll like receptor 4 (TLR4), together with its co-receptor CD14, plays a central role in innate immune response after engagement with lipopolysaccharide (LPS) during sepsis by triggering a signaling cascade for inflammation. In addition to LPS, several endogenous danger-associated molecular patterns (DAMPs), including the newly identified peroxiredoxin-1 (Prdx1), can also interact with CD14/TLR4 and elicit severe sepsis-like response [9–12].

Prdx1 is a member of the ubiquitous peroxiredoxin family proteins which possess antioxidant and chaperone activity [13]. In a rat hepatic ischemic model, Prdx1 expression and oxidation increase rapidly in liver cytosolic extract. Accordingly, a defensive role against oxidative stress in cells is suggested based on its peroxidase activity for scavenging endogenous reactive oxygen species (ROS) [14]. By contrast, extracellular release of Prdx1 could induce secretion of pro-inflammatory cytokines in rats with brain and lung injuries, hence it was considered a DAMP molecule [15, 16]. Recent studies have demonstrated that oxidation is a prerequisite for Prdx1 to be secreted by human embryonic kidney cells and monocytic cells in response to various stimuli [17]. Furthermore, proteomic analysis demonstrates that isolated mouse hearts release significant amount of Prdx1 into coronary effluent when exposed to I/R [18]. Thus, we hypothesized that Prdx1 arises in circulation of cardiogenic shock patients who are subjected to whole body I/R following ECMO intervention and instigates the systemic inflammation. In this study, we investigated the early kinetic changes of plasma Prdx1 and inflammatory cytokine levels in a cohort of cardiogenic shock patients undergoing ECMO. Indeed, there is a close link between Prdx1 and inflammatory cytokines which correlated to poor clinical outcomes. This result demonstrates that Prdx1 serves as an early biomarker for the cardiogenic shock patients who receive ECMO support.

## Methods

### Study population and outcome definition

Adult patients who were admitted to National Taiwan University Hospital, a university affiliated tertiary care center, for cardiogenic shock or cardiac arrest requiring ECMO support were prospectively enrolled from January 2010 to June 2013. ECMO was indicated for circulatory collapse or needing more than 40 inotropic equivalents (= dopamine + dobutamine + epinephrine × 100 + norepinephrine × 100, µg/kg/min) support [19]. The ECMO system (Medtronic Inc, Minneapolis, MN) was set up through femoral veno-arterial route. Exclusion criteria included pre-existing MOF and severe brain insult before ECMO. All-cause mortality within 7 days after ECMO initiation was defined as the primary end point. The survival time of more than 7 days was selected to focus on the rescue effect of cardiogenic shock by ECMO, and avoid the complications after the rescue. Development of MOF is often regarded as irreversible damages of cardiogenic shock [20]; therefore, all-cause mortality or MOF incompatible with life within 7 days after ECMO installation was selected as secondary end point, and the survival time for those who developed MOF within 7 days after ECMO support was defined as day 7 for survival analysis although they survived more than 7 days. The local research ethics committee approved this study protocol (Internal Review Board number 200911043R), and written informed consents were obtained from the closest relatives of the recruited patients.

### Blood sampling

Peripheral blood samples were withdrawn from patients upon ECMO installation before resuscitation (0 h) and 2, 6, 12, 24 h after oxygenation. Blood samples were collected into ethylenediamine tetraacetic acid (EDTA) containing tubes (Vacutainer, Becton–Dickinson, San Jose, CA), used immediately for flow cytometry analysis, or centrifuged at 2000*g* for 20 min at 4 °C to collect plasma, which were aliquoted and stored at −80 °C until analysis.

### ROS determination

The blood collected in a sodium heparin containing tube (Becton–Dickinson) was kept on ice and analyzed within 1 h. Total ROS content was measured by reacting 0.2 mL of blood with 1 mL of 0.3 mM luminol (Sigma-Aldrich, St. Louis, MO) for 4 min at 37 °C in a chemiluminescence analyzer (Tohoku Electronic Industrial Co. Ltd., Miyagi, Japan). Two measurements with their value difference less than 30 % were taken to calculate the average for the ROS production. ROS level was expressed as photon counts per minute (cpm).

### Flow cytometry

One hundred microliters of EDTA anticoagulated whole blood was mixed with the following mouse-anti human antibodies (BD Biosciences, San Jose, CA): 10 μL of phycoerythrin (PE) conjugated anti-CD14, 10 μL of fluorescein isothiocyanate (FITC) conjugated anti-CD16, and 12 μL of biotinylated anti-TLR4 antibodies. After incubation for 20 min at room temperature in the dark, red blood cells were lysed by 1.5 mL of BD lysing buffer, and white blood cells were washed twice with 1.5 mL of PBS containing 1 % fetal bovine serum and 0.1 % sodium azide. After centrifugation, 5 μL of allophycocyanin (APC) conjugated streptavidin (BD Biosciences, San Jose, CA) was added into the tube containing biotinylated anti-TLR4 antibody and incubated for another 20 min. The cells were washed and fixed in 0.5 mL of PBS with 0.25 % paraformaldehyde and kept on 4 °C until analysis. Cells were also labelled with negative isotype control for PE-mouse IgG1. Flow cytometry data were acquired on BD Calibur flow cytometer, and analysis was performed using CellQuest software version 3.2. Neutrophils, lymphocytes and monocytes were identified based on their forward scatter/side scatter (FSC/SSC) dot plot profiling (Additional file 1: Figure S1A). Monocytes were further gated in an SSC/CD14^+^ dot plot while CD14 positive cells were identified as their CD14 expression levels were higher than the isotype-defined fluorescent background and cells with granulocytes scattering properties were excluded (Additional file 1: Figure S1B, C). CD14-PE (Additional file 1: Figure S1D) and TLR4-APC (Additional file 1: Figure S1E) were measured on CD14^+^ monocytes and expressed as geometric mean fluorescence intensity (MFI).

### Plasma analysis

Plasma Prdx1 levels were measured by a commercial enzyme-linked immunoadsorbent assay (ELISA) kits (Northwest Life Science Specialties, Vancouver, WA). Carbonic anhydrase IX (CA IX) ELISA kit was from R&D Systems (Minneapolis, MN). Plasma cytokine levels were determined separately using ELISA kits for tumor necrosis factor-α (TNF-α), interleukin (IL)-6, IL-8, IL-10 (BD Biosciences), IL-1β and monocyte chemotactic protein (MCP)-1 (eBioscience, San Diego, CA).

### Cell culture treatment

Human peripheral blood mononuclear cells (PBMCs) were isolated from healthy volunteers using BD Vacutainer Cell Preparation Tubes. Monocytes were isolated from PBMCs by plastic adherence method [21], and cultured in RPMI 1640 medium (Gibco, Grand Island, NY) with 10 % fetal bovine serum (FBS) (Gibco) and 100 U/mL penicillin and 100 g/mL streptomycin. Human macrophages were obtained by culturing the monocytes with 100 ng/mL of macrophage-colony stimulating factor (BioLegend, San Diego, CA) for 4 days. For human monocyte derived dendritic cells (HMDCs), monocytes were purified from PBMCs by magnetic cell sorting using CD14 microbeads according to the manufacturer’s instructions (AutoMACS Pro Separator, Miltenyi Biotec, Teterow, Germany), followed by culturing the monocytes with 20 ng/mL of granulocyte macrophage-colony stimulating factor (PeproTech, Rocky Hill, NJ) and 20 ng/mL of IL-4 (eBioscience, San Diego, CA) for 5 days. Mouse macrophage cell line (RAW264.7) was maintained in DMEM medium (Gibco) containing 10 % FBS and antibiotics (penicillin/streptomycin). Human recombinant Prdx1 (Abnova, Taipei, Taiwan) at 11 μg/mL, LPS (*Escherichia coli* serotype 0111:B4, Sigma-Aldrich, St. Louis, MO) at 50 pg/mL, Polymyxin B (PMB, Sigma-Aldrich) at 10 μg/mL, human and mouse recombinant IFN-γ (R&D Systems) at 20 U/mL, rabbit polyclonal antibody to Prdx1 (ab59538, Abcam, Cambridge, MA) at 2.5 and 10 μg/mL and polyclonol rabbit IgG (AB-105-C, R&D system) at 10 μg/mL were added to the corresponding cells as described in the figures.

### Hypoxic treatment

Human monocytes were incubated in a humidified hypoxic chamber (Proox Model 110, Biospherix, Lacona, NY) and exposed to hypoxia (5 % CO_2_ level and less than 1 % O_2_ balanced with N_2_) for 18 or 6 h and followed by oxygenation for 24 h. Ambient oxygen concentrations of 1 % were maintained using a controlled incubator with CO_2_/O_2_ monitoring and CO_2_/N_2_ gas sources. Cells incubated at 5 % CO_2_ in a humidified atmosphere with 18 % O_2_ were used as normoxic controls.

### Statistical analysis

The data were analyzed using SPSS 17.0 (SPSS Inc, Chicago, IL). Owing to the non-normal distribution characteristic of most data, the medians and interquartile ranges were presented in some figures. Continuous data were also expressed as mean ± standard error of mean (SEM) in figures when appropriate. The significant differences between groups were compared by Mann–Whitney U test or Student’s *t* test. Categorical data were presented as percentages of patients and compared using *Χ*^2^ test or Fisher’s exact test. The correlations were analyzed by the Spearmen’s Rho test. Receiver-operating characteristic (ROC) analysis was performed to determine the optimal cut-off values for predicting the primary endpoint of 7-day all-cause mortality. The Kaplan–Meier survival curve was used to show the survival differences between different groups and the log-rank test was used to calculate the statistical significance.

## Results

To study the connection between Prdx1 levels and survival outcomes in victims afflicted with severe cardiovascular events, 46 patients who necessitated advanced circulation support by ECMO were enrolled. Of the 32 survivors, 20 patients survived to hospital discharge; 7 patients were diagnosed with MOF within 7 days of ECMO support, and their survival times ranged from 10 days to 71 days; the other 5 patients died of stroke, acute humoral rejection and sepsis-related MOF at day 8 to day 133. Fourteen patients died mainly from MOF within 7 days of ECMO onset. The baseline characteristics of these patients are shown in Table 1. There was no difference between survival and death groups for age, gender, risk factors such as coronary artery disease, diabetes, smoking, hypertension, diagnosis, and blood white cell categories.

**Table 1:** Demographic and clinical characteristics of the patients in this study according to their survival status at 7th day after receiving extracorporeal membrane oxygenation support

Variable	Survival	Death	*P* value
Sample size (*n*)	32 (69.6 %)	14 (30.4 %)	
Age (years) (*n* = 46)	51.5 (41.5–58.0)	54.7 (48.2–68.2)	0.118
Gender			1.000
Male (*n* = 35)	24 (68.6 %)	11 (31.4 %)	
Female (*n* = 11)	8 (72.7 %)	3 (27.3 %)	
Risk factors			
Hypertension (*n* = 21)	14 (43.8 %)	7 (50.0 %)	0.755
DM (*n* = 13)	7 (21.9 %)	6 (42.9 %)	0.171
CAD (*n* = 11)	6 (18.8 %)	5 (35.7 %)	0.269
Smoking (*n* = 9)	7 (21.9 %)	2 (14.3 %)	0.701
Dialysis (*n* = 5)	1 (3.1 %)	4 (28.6 %)	0.078
Pre-ECMO CPR (*n* = 13)	10 (31.3 %)	3 (21.4 %)	0.724
CPR during ECMO (*n* = 20)	11 (34.4 %)	9 (64.3 %)	0.105
Diagnosis			
AMI (*n* = 24)	16 (50.0 %)	8 (57.1 %)	0.754
DCMP (*n* = 12)	7 (21.9 %)	5 (35.7 %)	0.467
Myocarditis (*n* = 6)	6 (18.8 %)	0 (0.0 %)	0.157
Other (*n* = 4)	3 (9.4 %)	1 (7.1 %)	1.000
Heart rate (BPM) (*n* = 42)	116 (88–138)	96 (66–121)	0.196
Systolic blood pressure (mmHg) (*n* = 40)	90 (74–101)	93 (76–113)	0.902
Diastolic blood pressure (mmHg) (*n* = 40)	57 (47–67)	53 (44–64)	0.435
pH (*n* = 45)	7.41 (7.30–7.48)	7.23 (7.13–7.39)	0.066
PaCO_2_ (mmHg) (*n* = 45)	32 (23–39)	35 (27–46)	0.455
PaO_2_ (mmHg) (*n* = 45)	83 (56–156)	108 (60–222)	0.313
HCO_3_ (mmol/L) (*n* = 45)	19.3 (15.9–24.0)	17.6 (12.7–19.3)	0.107
Lactate (mmol/L) (*n* = 43)	6.7 (3.0–9.7)	10.9 (3.3–18)	0.260
Inotropic equivalent (μg/kg/min) (*n* = 34)	17.5 (8.2–25.2)	23.3 (18.7–34.4)	0.077
WBC (K/µL) (*n* = 46)	12.9 (8.7–15.4)	12.3 (9.1–18.4)	0.645
Granulocytes (% of total WBC) (*n* = 46)	72.8 (40.1–82.1)	60.0 (42.2–80.8)	0.856
Lymphocytes (% of total WBC) (*n* = 46)	17.3 (9.0–51.5)	34.2 (11.1–50.1)	0.612
Monocytes (% of total WBC) (*n* = 46)	4.0 (3.2–6.6)	3.9 (2.8–6.4)	0.772
CD14^+^ CD16^+^ (% of monocytes) (*n* = 46)	64.4 (46.4–82.2)	60.7 (43.7–71.6)	0.314

Values are medians with inter-quartile ranges for continuous data and frequency (percentage,  %) for categorical data. The listed *P* values of statistical tests were calculated using Mann–Whitney U test for continuous data and the *Χ* ^2^ or Fisher’s exact test for categorical data

*DM* diabetes mellitus, *CAD* coronary artery disease, *CPR* Cardiopulmonary resuscitation, *AMI* acute myocardial infarction, *DCMP* dilated cardiomyopathy, *BPM* beat per minute, *WBC* white blood cell

### Prdx1 levels and hypoxia

**Fig. 1:**
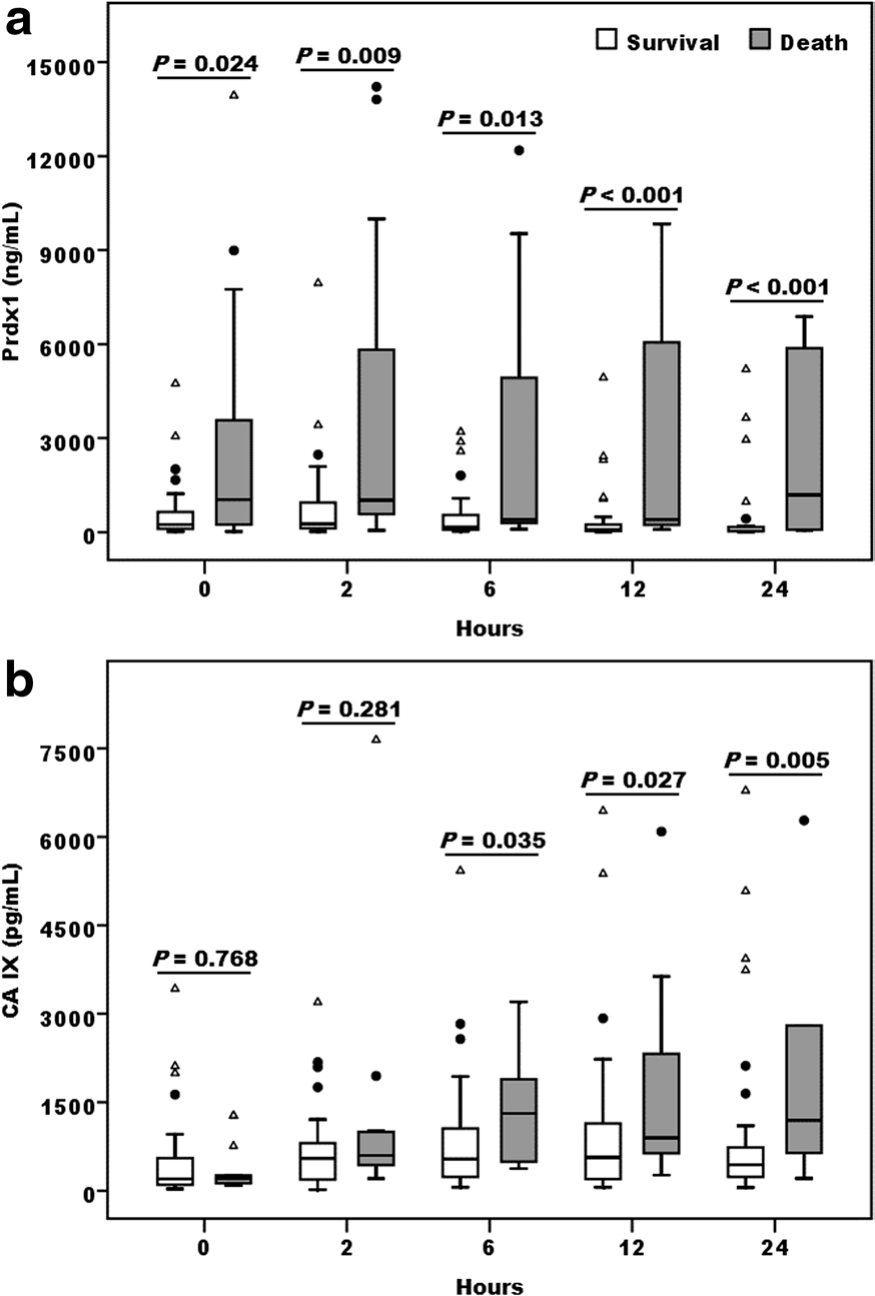
Peroxiredoxin 1 was an early phase predictor for the clinical outcomes of cardiogenic shock patients who received ECMO resuscitation. Plasma Peroxiredoxin 1 (Prdx1) (**a**) and carbonic anhydrase IX (CA IX) (**b**) levels in the cardiogenic shock patients at the indicated time points after receiving ECMO support according to their 7-day survival status [survivors (n = 32) vs. non-survivors (n = 14)]. Data were expressed as median and interquartile range, while *solid circle* and *open triangle* indicated mild and extreme outliers respectively. Statistical differences between groups were analyzed by Mann–Whitney U test

**Fig. 2:**
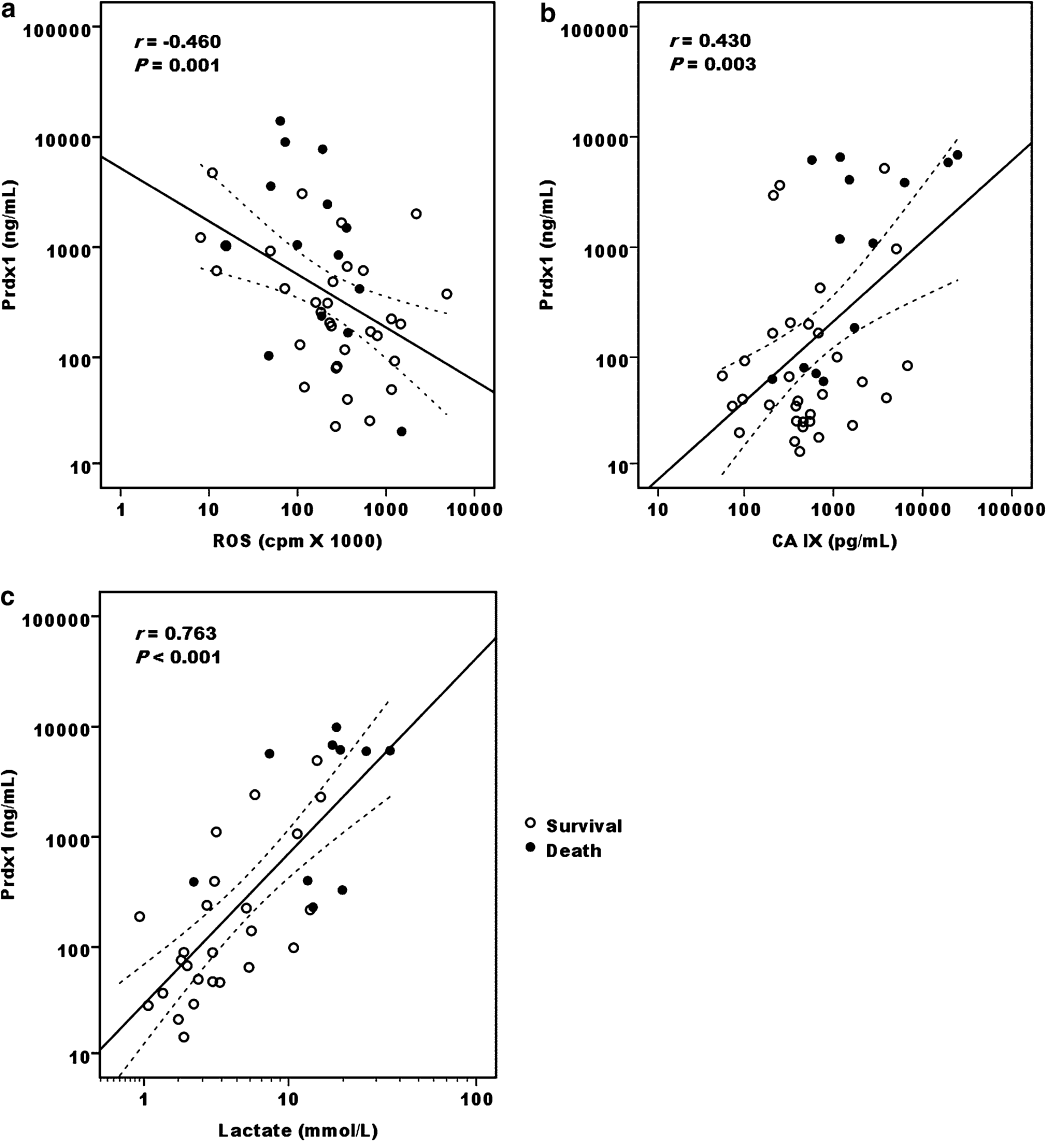
Correlation between Prdx1 and hypoxic markers. Plasma Prdx1 levels in the patients cohort as described in Fig. 1 were negatively related to ROS levels (**a**) at 0 h, but positively related to plasma CA IX (**b**) at 24 h after receiving ECMO support. Prdx1 levels at 12 h was also positively related to blood lactate (**c**) measured at 16 h after ECMO initiation in 34 patients. The correlations were analyzed by the Spearmen’s Rho test, with *dashed lines* indicating the 95 % confidence interval

Initial Plasma Prdx1 level in the death was 4.3-fold higher as that in survival (median 1036.2 vs. 240.8 ng/mL). Prdx1 in the non-survivors slightly decreased at 6 and 12 h, and then peaked at 24 h. In contrast, Prdx1 level in the survivors gradually declined after 2 h of ECMO installation. Thereby, the Prdx1 levels between these two groups were significantly different throughout the observational period (Fig. 1a). A hypoxic marker, the plasma CA IX, increased in both groups at 2 h when the CA IX level in the survivors would reach a plateau, whereas, the CA IX level in the deaths increased further at 6 h, and attained a significant difference thereafter compared to that in the survivors (Fig. 1b). Total ROS production showed no notable difference between the survivors and non-survivors at 0–24 h (Additional file 2: Figure S2). These results illustrated an early rapid release and implied a better prognostic performance of Prdx1 than ROS and CA IX in cardiogenic shock patients receiving ECMO support. Furthermore, the Prdx1 levels were negatively correlated with the ROS activities at 0 h (Fig. 2a) while a positive correlation between Prdx1 and CA IX concentrations was detected at later time point (Fig. 2b). In addition, the Prdx1 levels at 12 h had a strong correlation with the available blood lactate concentrations of 34 patients obtained at 16 h after ECMO installation (Fig. 2c). The persistently elevated Prdx1 and lactate levels could reflect severe tissue hypo-perfusion due to insufficient ECMO support. These findings are compatible with the consensus that plasma Prdx1 possesses ROS scavenging ability, and probably reflect the severity of tissue hypoxia in this patient cohort.

### Prdx1 and systemic inflammatory cytokines

**Fig. 3:**
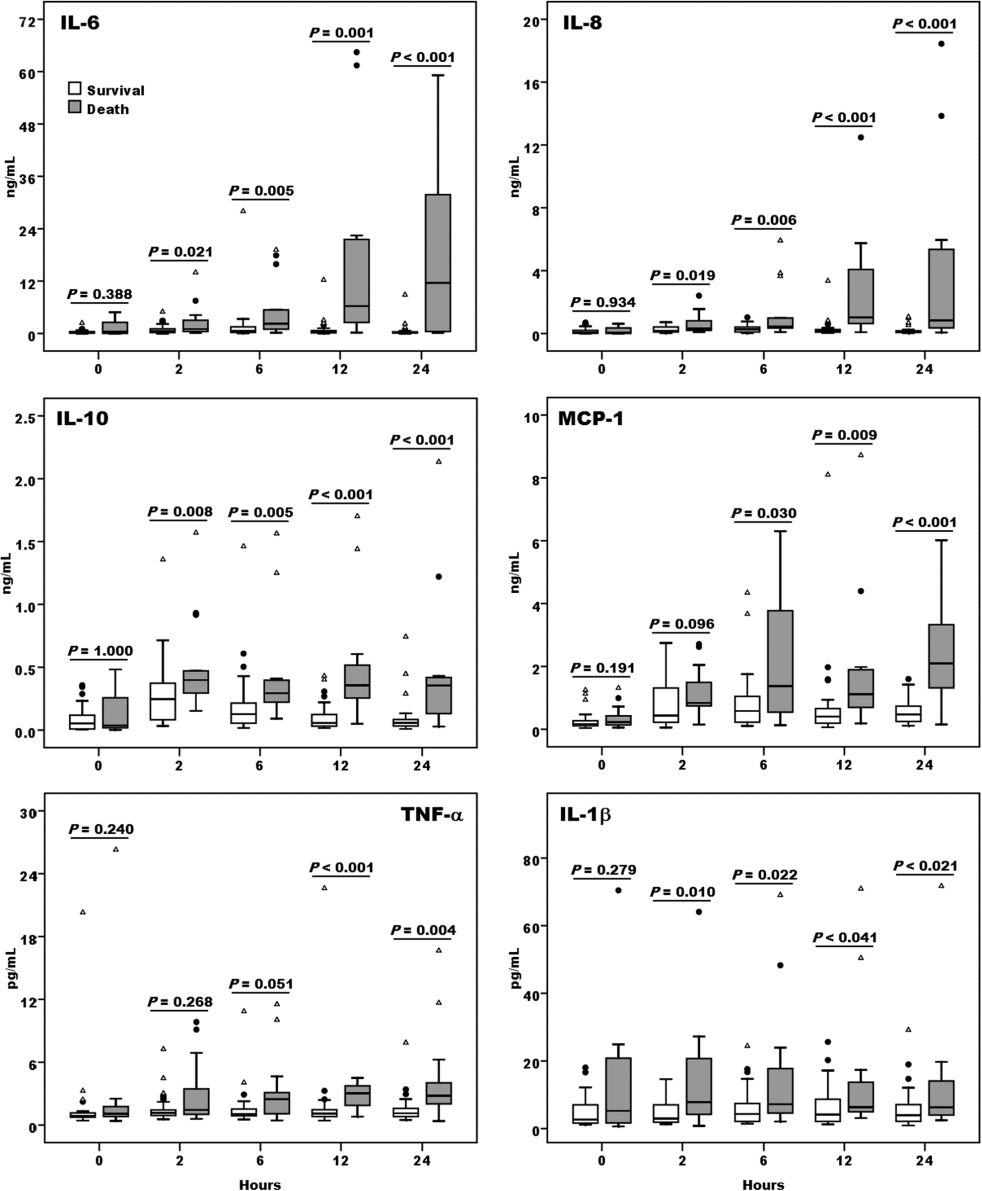
Comparison of inflammatory cytokine profiles between survivors and non-survivors who received ECMO support. Plasma IL-6, IL-8, IL-10, monocyte chemotactic protein-1 (MCP-1), tumor necrosis factor-α (TNF-α), and IL-1β levels were assessed among survivors (n = 32, *open boxes*) and non-survivors (n = 14, *grey boxes*) at the indicated time points after implement of ECMO support. Data represented the median and interquartile range of each group, while *solid circle* and *open triangle* indicated mild and extreme outliers respectively. Statistical differences between groups were analyzed by Mann–Whitney U test

**Fig. 4:**
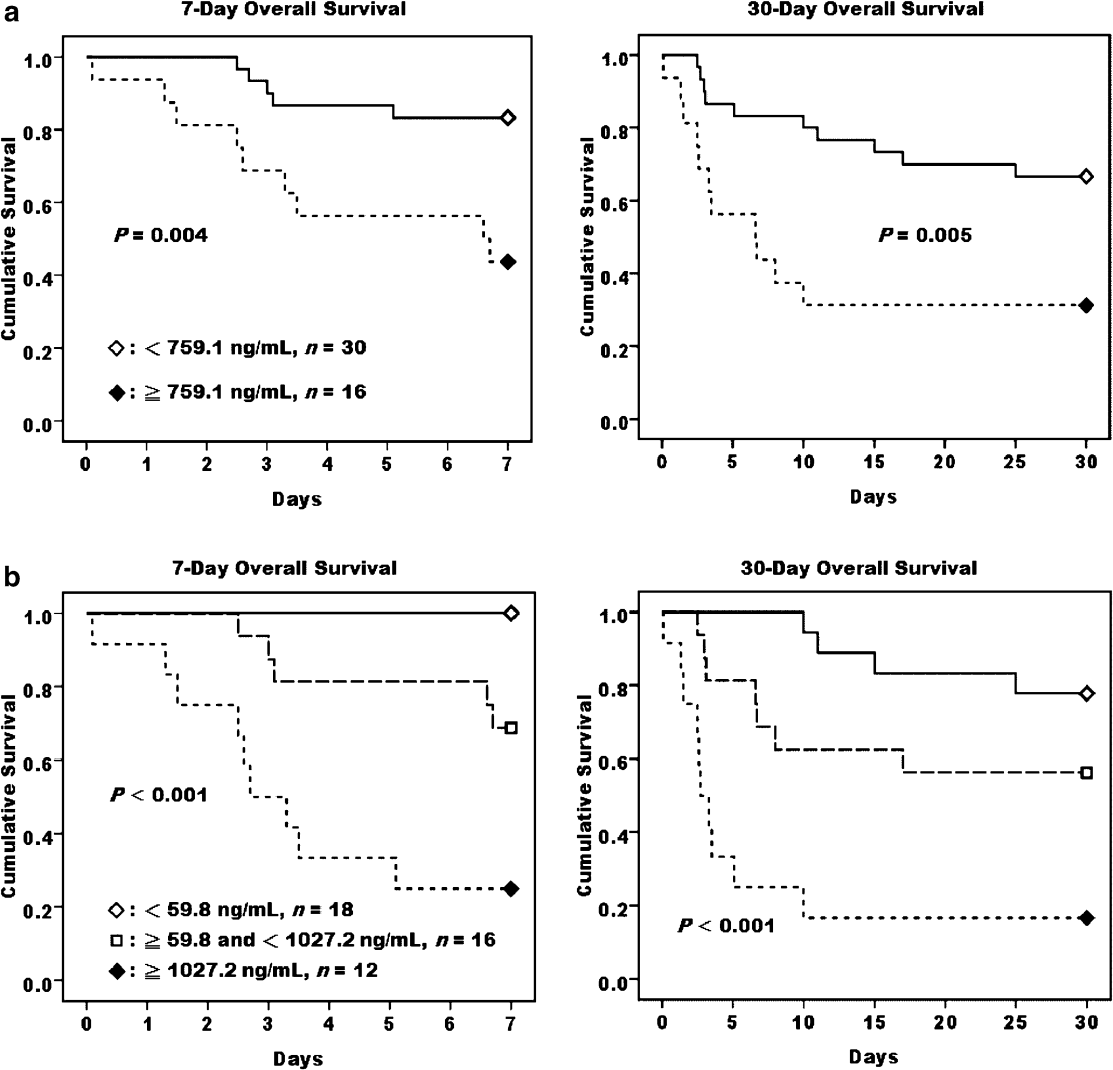
High initial Peroxiredoxin 1 level in cardiogenic shock patients who received ECMO support was associated with a poorer survival. Kaplan–Meier survival analysis of 7- and 30-day overall survivals in cardiogenic shock patients categorized by the cut-off values, as presented in Table 2, of their plasma Prdx1 levels at 0 (**a**) and 24 h (**b**) after receiving ECMO support

We then compared the plasma levels of TNF-*α*, IL-1*β*, IL-6, IL-8, IL-10 and MCP-1 in these two groups of patients. These inflammatory cytokine levels in the death group became significantly higher than the survivors as early as 2 h (Fig. 3). ROC analysis (Additional file 3: Figure S3A, B) demonstrated that only Prdx1, but not the other cytokine levels at 0 h could differentiate all-cause mortality outcomes on day 7 of these patients, while Prdx1 and cytokine levels at 24 h after ECMO support were both associated with worse outcomes (Table 2). It was worth notice that two optimal cut-off values of plasma Prdx1 levels at 24 h were obtained by Youden-index. To better differentiate the significance of Prdx1 levels as an early phase marker for the outcome of the patients recruited in current study, we performed Kaplan–Meier survival analysis. The result showed that plasma Prdx1 levels were very informative in predicting the eventual outcome of cardiogenic shock patients receiving ECMO support. The patients with Prdx1 levels lower than the optimal cut-off values at 0 h (Fig. 4a) and 24 h (Fig. 4b) had better outcomes in both the 7-day and 30-day overall survivals than the patients whose Prdx1 levels were higher than those cut-off values. Similarly, patients with lower initial Prdx1 had better chance of maintaining event free survivals during a short term (7-day) and a long term (30-day) observation period (Additional file 4: Figure S4A, B). Correlation analysis revealed that plasma Prdx1 measured at 2–24 h correlated well with all the cytokines except IL-1*β* at the corresponding time points (Table 3). This result inferred that plasma Prdx1 could be an upstream inducer of these cytokines.

**Table 2:** Predictive values of peroxiredoxin 1 and cytokines for 7-day all cause mortality rate

Biomarkers	Cut-off (ng/mL)	AUC	SE	95 % CI	Sensitivity	Specificity	*P* value
0 h							
Prdx1	759.09	0.734	0.092	0.56–0.91	0.692	0.774	0.015
CA IX	0.123	0.531	0.091	0.35–0.71	0.769	0.419	0.748
IL-6	0.839	0.618	0.112	0.40–0.84	0.462	0.903	0.222
IL-8	0.333	0.524	0.101	0.33–0.72	0.308	0.839	0.807
IL-10	0.245	0.526	0.103	0.32–0.73	0.308	0.903	0.787
MCP-1	0.211	0.618	0.098	0.43–0.81	0.615	0.677	0.222
TNF-α	0.0013	0.653	0.090	0.48–0.83	0.462	0.839	0.114
IL-1β	0.019	0.600	0.104	0.40–0.81	0.308	1.000	0.298
24 h							
Prdx1	59.78	0.861	0.059	0.75–0.98	1.000	0.567	<0.001
	1027.23				0.667	0.900	
CA IX	0.466	0.811	0.065	0.68–0.94	1.000	0.567	0.002
IL-6	0.984	0.883	0.062	0.75–1.00	0.750	0.900	<0.001
IL-8	0.208	0.881	0.064	0.75–1.00	0.917	0.800	<0.001
IL-10	0.083	0.861	0.071	0.72–1.00	0.917	0.767	<0.001
MCP-1	1.223	0.856	0.088	0.00–1.00	0.833	0.933	<0.001
TNF-α	0.0020	0.828	0.076	0.68–0.98	0.833	0.833	0.001
IL-1β	0.0043	0.736	0.081	0.58–0.90	0.667	0.700	0.018

Cut-off values are determined by receiver-operating characteristic (ROC) analysis

*Prdx* peroxiredoxin, *CA* carbonic anhydrase, *IL* interleukin, *MCP* monocyte chemotactic protein, *TNF* tumor necrosis factor, *AUC* area under the ROC curve, *SE* standard error, *CI* confidence interval

**Table 3:** Correlations between plasma peroxiredoxin 1 and cytokines in cardiogenic shock patients at the indicated time points after receiving extracorporeal oxygenation membrane support

Prdx1	0 h	2 h	6 h	12 h	24 h
IL-6	0.155	0.637^**^	0.612^**^	0.648^**^	0.616^**^
IL-8	−0.002	0.481^**^	0.565^**^	0.650^**^	0.653^**^
IL-10	−0.026	0.573^**^	0.571^**^	0.704^**^	0.684^**^
MCP-1	0.098	0.425^**^	0.553^**^	0.463^**^	0.600^**^
TNF-α	0.337^*^	0.476^**^	0.373^**^	0.599^**^	0.710^**^
IL-1β	0.052	0.187	0.330^*^	0.251	0.179

Values are Spearman’s correlation coefficients, ^*^ *P* < 0.05 and ^**^ *P* < 0.01, respectively

*Prdx* peroxiredoxin, *IL* interleukin, *MCP* monocyte chemotactic protein, *TNF* tumor necrosis factor

### TLR4 signaling activation

**Fig. 5:**
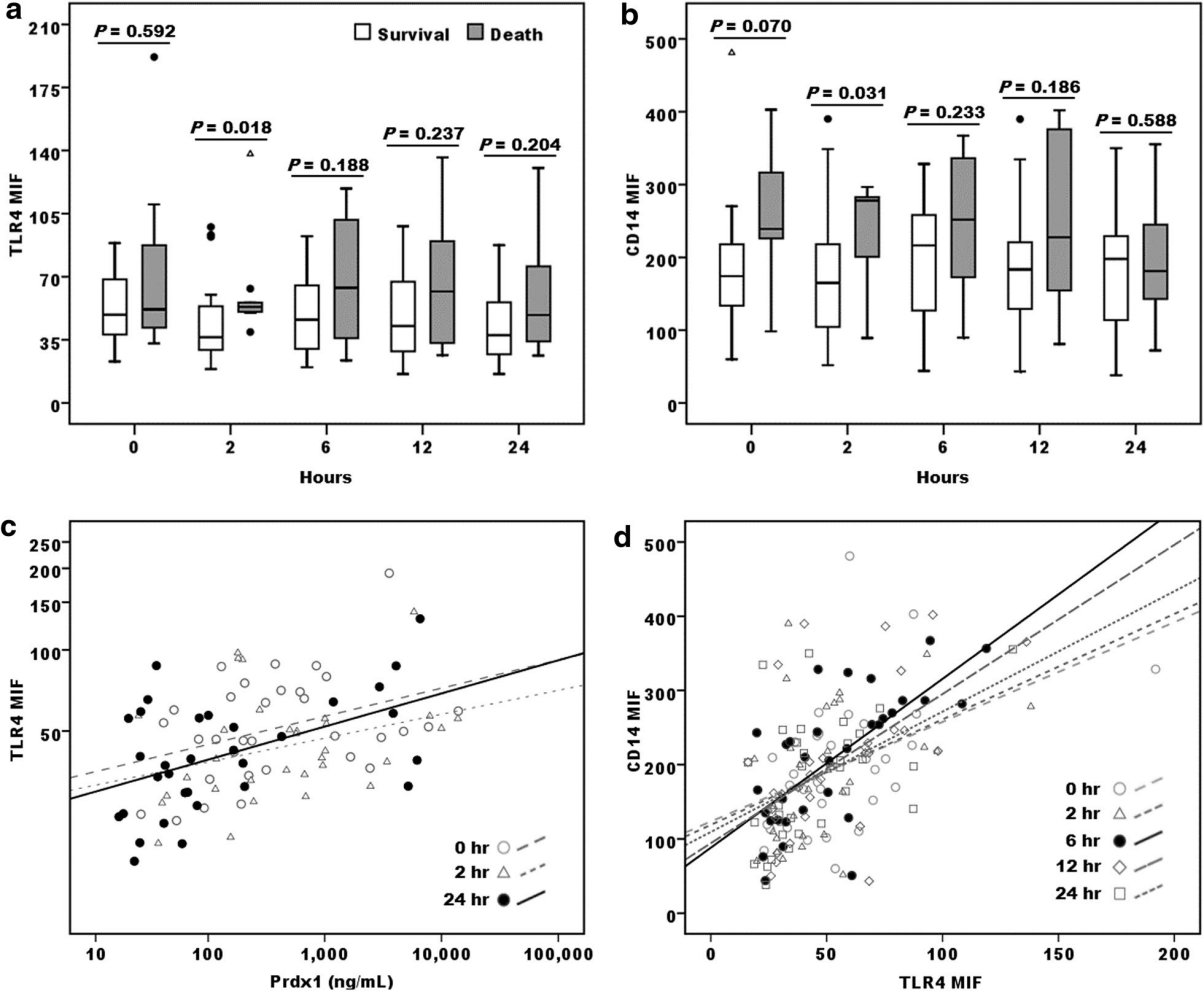
Interaction of TLR4, CD14 and Prdx1 in cardiogenic shock patients who received ECMO support. Monocyte TLR4 (**a**) and CD14 (**b**) surface expressions [median and interquartile range of the mean fluorescence intensity (MFI) as assessed by flow cytometry analysis] at the indicated time points in 24 survivors (*open boxes*) and 9 deaths (*grey boxes*). **c** TLR4 expression on monocytes was found best correlated to the Prdx1 levels at 24 h after initiating ECMO support (correlation coefficient = 0.425, *P* = 0.015, indicated by *solid circle* and *line*). Similarly, plasma Prdx1 levels at 0 and 2 h were also noted to be positively related (indicated by dotted lines). **d** TLR4 and CD14 expressions were found positively correlated to each other at all the time points through the observation course within 24 h. The *dotted lines* indicated the linear fits for the correlation of both variables at each time point. *Solid circles* and line indicated 6 h after initiating ECMO support was the time point with the strongest correlation between TLR4 and CD14 expression on the monocytes of patients (correlation coefficient = 0.699, *P* < 0.001)

We measured TLR4 and CD14 expressions of the last 33 consecutive patients we recruited in this study. The surface expression levels of TLR4 and CD14 on the monocytes of the death group were significantly higher at 2 h than those in the survivors, and this difference seemed to level off at later time points (Fig. 5a, b). Intriguingly, plasma Prdx1 concentrations examined at 0, 2 and 24 h correlated positively to TLR4 expression levels on monocytes at the corresponding time points (Fig. 5c), indicating a functional relationship between Prdx1 and its receptor system evolved during the early phase of patients with severely compromised cardiovascular function. In addition, a positive correlation between monocyte TLR4 and CD14 levels was also noted throughout the course of observation (Fig. 5d). Taken together, Prdx1 might trigger or stabilize a formation of complexes with its receptor and co-receptor on the cellular membrane of its target cells.

### rPrdx1 induced cytokine secretions from IFN-γ priming macrophages

**Fig. 6:**
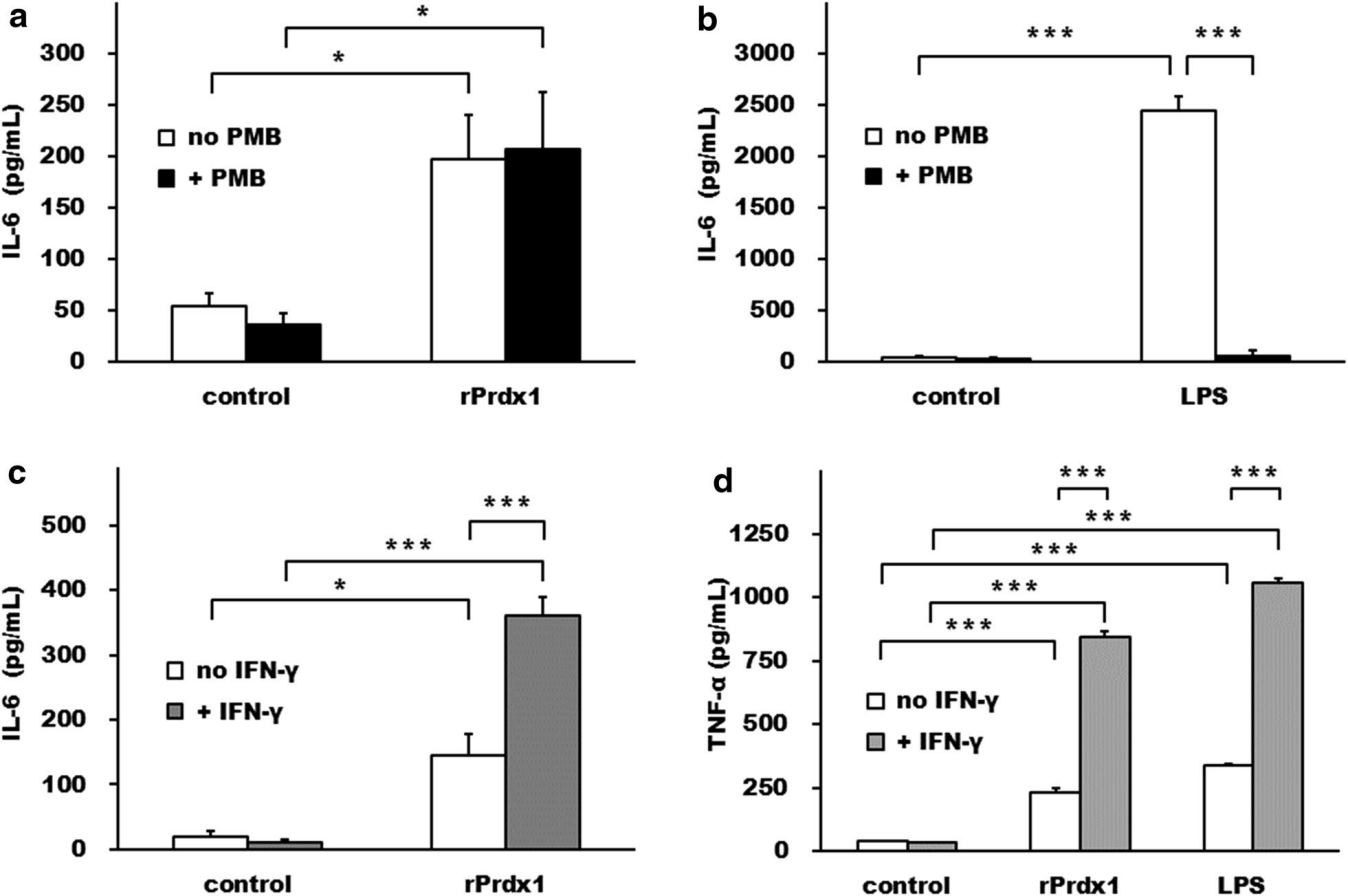
Prdx1 induce IL-6 and TNF-α production in INF-γ primed macrophage. Human recombinant Prdx1 (rPrdx1) at 11 μg/mL (**a**) or LPS (50 pg/mL) (**b**), that was either pretreated with PMB (10 μg/mL) or not, was added to human monocyte derived dendritic cells for 24 h before the culture supernatants were collected and analyzed for IL-6 levels. INF-γ (20 U/mL) pretreated human monocyte derived macrophages (**c**) or mouse macrophage RAW264.7 cells [12] were incubated with Prdx1 (11 μg/mL) or LPS (50 pg/mL) for 24 h before the supernatants were harvested and analyzed for IL-6 (**c**) or TNF-α [12] levels. Solvent vehicles diluted in the culture media were used as the* negative* controls. Data represented means of triplicate samples; *error bars* represented SEMs. *P < 0.05, and ***P < 0.001

Given the above results, we hypothesized that Prdx1 could induce cytokine production. To investigate this possibility, recombinant human Prdx1 (rPrdx1) was added to HMDCs. Indeed, IL-6 was moderately induced (Fig. 6a) although not to the extent as elicited by LPS (Fig. 6b). This result was not due to a contamination of bacterial endotoxin during preparation of the recombinant protein, because PMB did not block this effect. In contrast, the IL-6 induced by LPS could be significantly inhibited by PMB (Fig. 6a, b). rPrdx1 also induced IL-6 secretion from human macrophages, and the effect was augmented by pre-incubation of the cells with IFN-γ for 18 h (Fig. 6c). This IFN-γ-priming effect was also evident in rPrdx1 or LPS-induced TNF-α secretion from mouse macrophage RAW264.7 cells (Fig. 6d).

### Hypoxia enhanced rPrdx1-induced cytokine secretions from monocytes

**Fig. 7:**
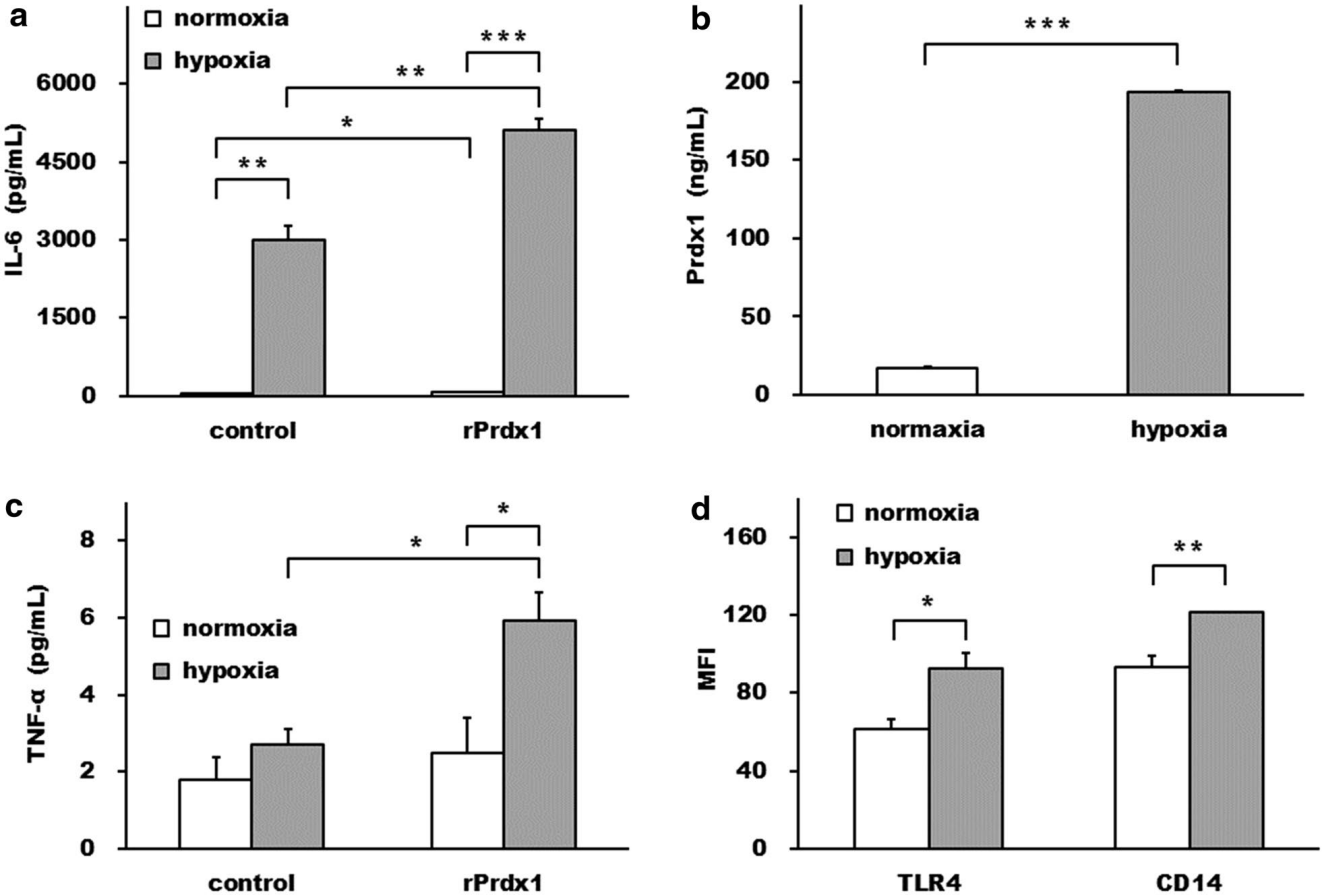
Hypoxia enhanced Prdx1-induced IL-6 and TNF-α production. Human monocytes, cultured under normoxia or hypoxia condition for 18 h, were incubated either with solvent control or rPrdx1 (11 μg/mL) for another 24 h in normoxia before the supernatants were harvested and analyzed for IL-6 (**a**) and TNF-α (**c**) levels. **b** The culture supernatants from human monocytes, under normoxia or hypoxia condition for 18 h and then followed by another 24 h in normoxia, were harvested and analyzed for the Prdx1 levels. **d** Human monocytes, cultured under normoxia or hypoxia condition for 6 h, were further incubated in normoxia for 24 h before surface TLR4 and CD14 expressions were analyzed. Data represented means and SEM for triplicate samples. *P < 0.05, **P < 0.01, and ***P < 0.001

In order to determine the role of hypoxia/reoxygenation in cytokine production by Prdx1, we pretreated human monocytes with hypoxia (1 % O_2_) for 18 h followed by normoxia for 24 h in the absence or presence of rPrdx1. As shown in Fig. 7a, rPrdx1 induced a marginal IL-6 secretion from monocytes, but this effect could be greatly augmented by hypoxia/reoxygenation. Although increased IL-6 secretion was also observed in the absence of rPrdx1 addition (Fig. 7a), the release of endogenous Prdx1 induced by hypoxia/reoxygenation treated monocytes may account for this elevation of IL-6 (Fig. 7b). rPrdx1 had insignificant effect on TNF-α secretion in monocytes under normoxia, whereas it was able to induce TNF-α secretion from hypoxia/reoxygenation treated monocytes (Fig. 7c). Reoxygenation for 24 h following a shorter period of hypoxia treatment (6 h) induced TLR4 and CD14 expressions on monocytes (Fig. 7d).

### Prdx1 stimulated IL-6 secretion under hypoxia/reoxygenation treatment

**Fig. 8:**
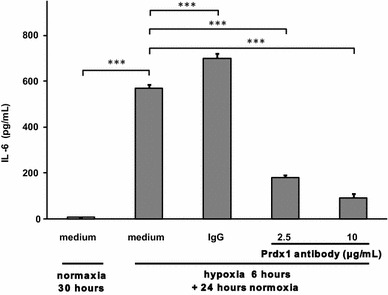
Prdx1 inhibition blocked hypoxia/reoxygenation-induced IL-6 secretion. After hypoxia treatment for 6 h, control rabbit IgG (10 μg/mL) and Prdx1 antibody (2.5 and 10 μg/mL) were added separately to human monocytes under normoxia condition for another 24 h before the supernatants were collected and analyzed for IL-6 levels. Data represented means and SEM for triplicate samples. ***P < 0.001

Although IL-6 secretion increased after hypoxia/reoxygenation in monocytes (Fig. 7a) and hypoxia also induced Prdx1 (Fig. 7b), the physiological significance of Prdx1 in the induction of IL-6 during hypoxia remains elusive. To address the definitive role of Prdx1 in hypoxia-induced production of IL-6 and explore the potential therapeutic possibility, we applied Prdx1 specific antibody to monocytes after hypoxic challenge. As seen in Fig. 8, Prdx1 antibody at 2.5–10 μg/mL, but not the control immunoglobulin, drastically inhibited the secretion of IL-6. This result reinforced our previous conclusion that the IL-6 released by hypoxia/reoxygenation treated human monocytes is due to the stimulation of Prdx1, and thus demonstrated that Prdx1 might serve as a potential therapeutic target aimed at alleviating cytokine storm under severe tissue hypoxia situation such as those cases receiving ECMO supports.

## Discussion

In the present study, we showed for the first time that plasma Prdx1 was linked to poor outcomes in patients with cardiogenic shock undergoing ECMO. Prdx1 was also correlated to blood lactate and plasma CA IX levels, possibly reflecting a profound and irreversible hypoxic damage occurring in the non-survivors. Like other DAMPs, Prdx1 in circulation can be released from necrotic cells and/or secreted actively by stressed cells [22, 23]. Additionally, it was shown that only the oxidized Prdx1 can be secreted from cells encountering certain oxidant or inflammatory stimuli [24]. Thus, plasma Prdx1 may reflect the extent of oxidative stress. The inverse correlation between Prdx1 and ROS levels before the initiation of ECMO signifies that Prdx1 may intensely participate in the intracellular ROS scavenging for cardiogenic shock patients. The positive correlation between high initial Prdx1 level and early phase mortality rate in cardiogenic shock patients undergoing ECMO support is, however, surprising given the assumption that Prdx1 is a protective ROS scavenger [14]. A possible explanation for this paradox might rely on the biochemical nature of the Prdx1 found in the circulation of hypoxic patients. It has been reported that secretion of Prdx1 is through a redox-dependent mechanism involving cysteine oxidation [17]. Thereby, the high Prdx1 found in the plasma of non-survivors in our cohort might belong to a population of oxidized molecules which have already or temporarily lost their ROS scavenging capability. Therefore, further studies to identify the redox status of Prdx1 in plasma are mandatory for clarifying their extracellular roles beyond their established intracellular role as a ROS scavenger.

Overwhelming production of pro-inflammatory cytokines can activate interaction of circulating leukocytes and vascular endothelial cells, and result in leukocyte-dependent microvascular injury which is a major feature of I/R injury and MOF [4]. Though it is well recognized that prolonged ischemia causes tissue and organ damage, the injury induced by reperfusion may be even more detrimental. Our study found a rapid burst of various plasma cytokines within 2 h of ECMO regardless of the clinical outcomes, implying a release of pre-formed cytokines from tissue stores, as described in a porcine model with ECMO institution [25]. The half-lives of plasma cytokines are generally less than 2 h [26]. Hence the rising cytokine levels which persisted for 1 day in the non-survivors of this study may reflect the synthesis and continuous release of these de novo cytokines during the ECMO support. Consequently, plasma cytokine levels at 24 h could provide better prognostic information beyond that of early time points (Table 2). It is worth note that optimal cut-off values of Prdx1 at 0 h could differentiate the outcomes of both the 7 and 30 days overall and event-free survivals (Fig. 4; Additional file 4: Figure S4), implying that early measurements of Prdx1 in cardiogenic shock patients could provide prognostic information during the course of ECMO application.

The role of Prdx1 is not confined to its antioxidant activities. Interaction of ligand and receptor is the leading facet for the functional study of extracellular Prdx1 [27]. In vitro experiment demonstrates that binding of Prdx1 to TLR4 on mouse macrophages occurs within minutes and results in internalization of both molecules [12]. The internalization of Prdx1/TLR4 complex is similar to the endocytosis of LPS/TLR4 which is known to modulate multiple purposes of receptor signaling, including TLR4 recycling or degradation for signal sensitivity [28, 29]. As shown in Fig. 5, TLR4 expressions were higher in death group at 2 h, and this difference diminished at later time points, indicating a rapid dynamic change of this receptor on monocyte membrane after ECMO installation. It has been reported that CD14 is required for optimal cytokine secretions in response to Prdx1 [12] and is also essential for TLR4 endocytosis triggered by LPS [30, 31]. Accordingly, the finding of positive correlation between monocyte CD14 and TLR4 fluorescence intensities in this study can be regarded as a likely indication for ligand/receptor complex cooperation and internalization, which can also be seen in patients with on-pump coronary artery bypass surgery [32].

A good correlation between Prdx1 and cytokine levels at every time point after ECMO support (Table 3) evoked that circulating Prdx1 may be an important mediator in the development of ECMO-related SIRS. Our in vitro experiments proved that rPrdx1 indeed induced IL-6 and TNF-α release from macrophages. Moreover, IFN-γ priming together with rPrdx1 can further stimulate this inflammatory response, consolidating the evidence that Prdx1 acts as a pro-inflammatory initiator.

Although hypoxia is known to regulate the synthesis of Prdx1 [33], our finding that the release of endogenous Prdx1 from hypoxia/reoxygenation treated monocytes is novel. Several endogenous TLR4 ligands have been shown in patients with ischemic heart diseases that increase pro-inflammatory cytokine productions and are associated with disease severity [34–37]. Therefore, in order to testify whether Prdx1 is a previously unheralded danger signal in I/R injury, neutralization of Prdx1 using specific antibody was performed to examine the pro-inflammatory effect of Prdx1 (Fig. 8). Apparently, the dramatic inhibition of IL-6 secretion with neutralization antibody indicates that Prdx1 may play a critical role in the pathogenesis of I/R related SIRS by promoting inflammation and serve as a novel candidate for the development of therapies against I/R injury.

## Conclusions

For the first time, we discovered the early prognostic role of plasma Prdx1 in cardiogenic shock patients receiving ECMO and revealed the correlation of Prdx1 between innate immunity receptors and tissue hypoxia. In vitro investigation showed that Prdx1 might play a role in the regulation of proinflammatory cytokine expressions and could be a potential therapeutic target for treating ischemia/reperfusion injury.

## Additional files

10.1186/s12967-016-0869-xCD14 and TLR4 expressions on CD14+ monocytes analyzed by flow cytometry. (A) A representative forward (FSC) and side scattering (SSC) plot of lymphocytes (R1), monocytes (R2) and granulocytes (R3) was shown. (B) Isotype control antibody was used to establish the background fluorescence levels of monocytes (M1), and the percentage of positive cells was determined after staining of the cells with specific antibody (M2). (C) The CD14+ monocytes population was gated as in the SSC/CD14 dot plot. The CD14 (D) and TLR4 (E) expression levels were calculated as mean fluorescence intensity (MFI) of the populations as gated using the methodology described for panel B.
10.1186/s12967-016-0869-xROS levels in the cardiogenic shock patients who received ECMO support. Blood ROS levels were detected by luminol at the indicated time points after receiving ECMO support according to their 7-day survival status [survivors (n = 32) vs. non-survivors (n = 14)]. Data were expressed as median and interquartile range, solid circle and open triangle indicated mild and extreme outliers respectively. Statistical differences between groups were analyzed by Mann–Whitney U test.
10.1186/s12967-016-0869-xROC analysis for potential biomarkers in predicting outcome of patients with cardiogenic shock receiving ECMO support. ROC curves for plasma biomarkers measured at 0 hr (A) and 24 hr (B) after ECMO support to differentiate the 7-day mortality.
10.1186/s12967-016-0869-xHigh initial Peroxiredoxin 1 level in cardiogenic shock patients who received ECMO support was associated with a poorer event-free survival. Kaplan–Meier survival analysis of 7- and 30-day event free survivals in cardiogenic shock patients categorized by the cut-off values, as presented in Table 2. Event free survivals: survival status without multiple organ failure under the condition when assessment of sequential organ failure assessment (SOFA) score was lower than 15.

## Abbreviations

APC: allophycocyanin
CA IX: carbonic anhydrase IX
DAMP: danger-associated molecular pattern
ECMO: extracorporeal membrane oxygenation
EDTA: ethylenediamine tetraacetic acid
FBS: fetal bovine serum
FITC: fluorescein isothiocyanate
HMDC: human monocyte derived dendritic cell
IL: interleukin
I/R: ischemia/reperfusion
LPS: lipopolysaccharide
MCP: monocyte chemotactic protein
MFI: mean fluorescence intensity
MOF: multisystem organ failure
PBMC: peripheral blood mononuclear cell
PE: phycoerythrin
PMB: polymyxin B
Prdx1: peroxiredoxin1
ROC: receiver-operating characteristic
ROS: reactive oxygen species
SEM: standard error of mean
SIRS: systemic inflammatory response syndrome
TLR: toll like receptor
TNF: tumor necrosis factor
