# Supplementary figures and images for: Peroxiredoxin 1 induces inflammatory cytokine response and predicts outcome of cardiogenic shock patients necessitating extracorporeal membrane oxygenation: an observational cohort study and translational approach
Source: J Transl Med. 2016 May 4;14:114. doi: 10.1186/s12967-016-0869-x (PMC4855332; doi:10.1186/s12967-016-0869-x)

Figure S2


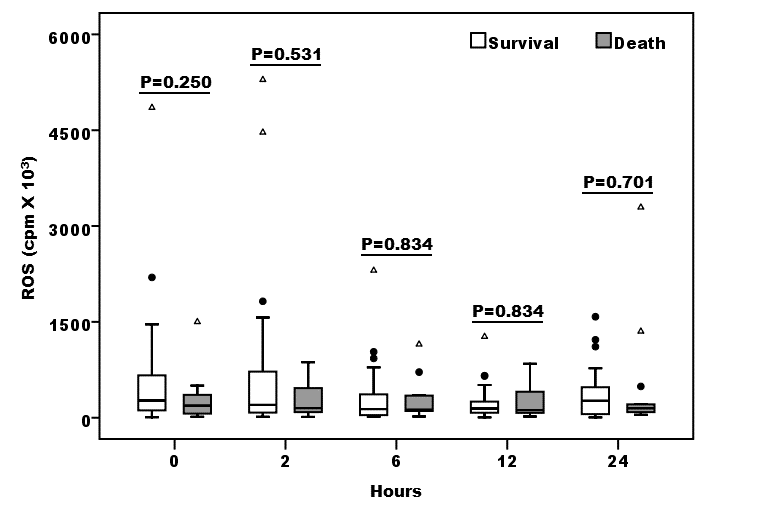

Supplement: Supplementary file 2 — 10.1186/s12967-016-0869-xROS levels in the cardiogenic shock patients who received ECMO support. Blood ROS levels were detected by luminol at the indicated time points after receiving ECMO support according to their 7-day survival status [survivors (n = 32) vs. non-survivors (n = 14)]. Data were expressed as median and interquartile range, solid circle and open triangle indicated mild and extreme outliers respectively. Statistical differences between groups were analyzed by Mann–Whitney U test. [file 12967_2016_869_MOESM2_ESM.docx]

Figure S3


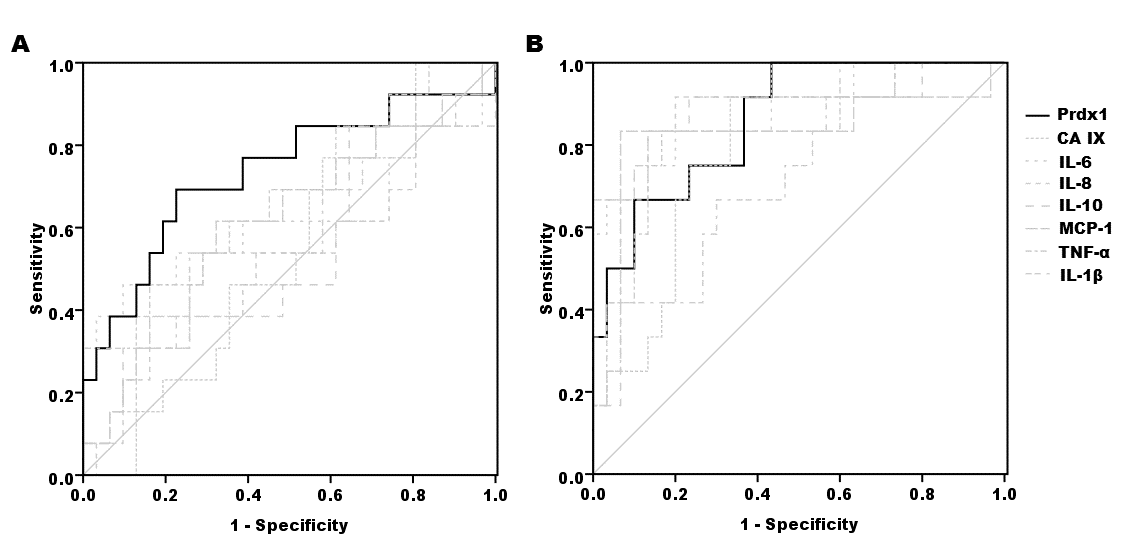

Supplement: Supplementary file 3 — 10.1186/s12967-016-0869-xROC analysis for potential biomarkers in predicting outcome of patients with cardiogenic shock receiving ECMO support. ROC curves for plasma biomarkers measured at 0 hr (A) and 24 hr (B) after ECMO support to differentiate the 7-day mortality. [file 12967_2016_869_MOESM3_ESM.docx]

Figure S4


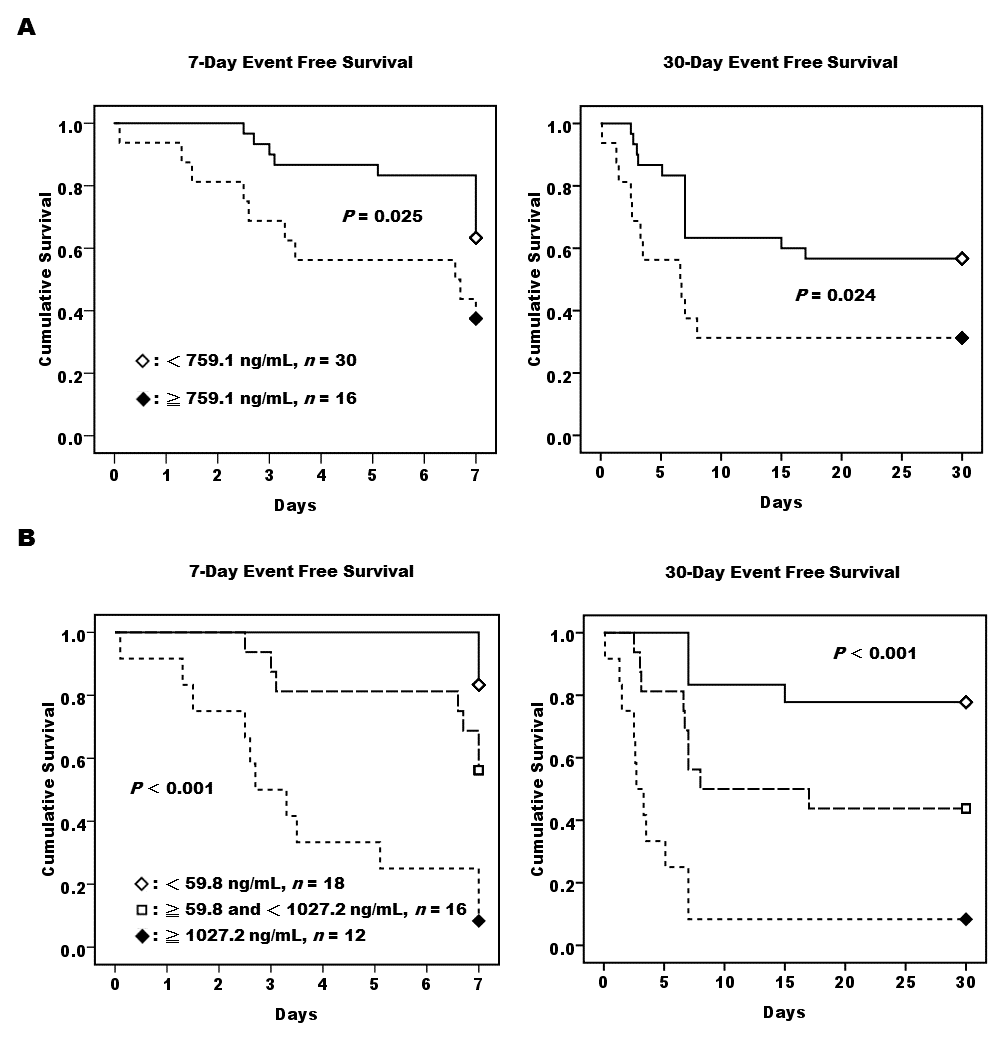

Supplement: Supplementary file 4 — 10.1186/s12967-016-0869-xHigh initial Peroxiredoxin 1 level in cardiogenic shock patients who received ECMO support was associated with a poorer event-free survival. Kaplan–Meier survival analysis of 7- and 30-day event free survivals in cardiogenic shock patients categorized by the cut-off values, as presented in Table 2. Event free survivals: survival status without multiple organ failure under the condition when assessment of sequential organ failure assessment (SOFA) score was lower than 15. [file 12967_2016_869_MOESM4_ESM.docx]
